# Crystal structure of (2-acetyl­ferrocen-1-yl)boronic acid

**DOI:** 10.1107/S2056989019001178

**Published:** 2019-01-29

**Authors:** Andrea Preuss, Marcus Korb, Heinrich Lang

**Affiliations:** a Technische Universität Chemnitz, Faculty of Natural Sciences, Institute of Chemistry, Inorganic Chemistry, D-09107 Chemnitz, Germany

**Keywords:** crystal structure, ferrocene, planar chirality, boronic acid, *ortho*-functionalization

## Abstract

(2-Acetyl­ferrocen-1-yl)boronic acid, [Fe(C_5_H_5_)(C_7_H_8_BO_3_)] or 2-C(O)CH_3_-1-B(OH)_2_–Fc [Fc = Fe(η^5^-C_5_H_3_)(η^5^-C_5_H_5_)], crystallizes in the centrosymmetric space group *P*2_1_/*n*. The boronic acid functionality inter­acts *via* intra­molecular hydrogen bonds with the acetyl group and with the –B(OH)_2_ functionality of an adjacent mol­ecule. The resulting centrosymmetric dimer exhibits an *anti*-positioning of the ferrocenyl moieties towards the central B_2_O_4_ plane. Consequently, an (*R_p_*,*S_p_*)-, *i.e.* a *meso* configuration is present for this dimer. In the crystal, weak C—H⋯O hydrogen bonds consolidate the mol­ecular packing.

## Abstract 2

In the crystal structure of (2-acetyl­ferrocen-1-yl)boronic acid, centrosymmetric dimers held together by –B(OH)⋯O hydrogen bonds are present.

## Chemical context   

The synthesis of 1,2-functionalized ferrocenes is a striking topic in ferrocene chemistry (Schaarschmidt & Lang, 2013 ▸; Korb *et al.*, 2014*a* ▸) and is mostly realized *via ortho*-directed metalation and subsequent reaction with electrophiles (Schaarschmidt & Lang, 2013 ▸) or intra­molecular rearrangement (Werner & Butenschön, 2013 ▸; Korb & Lang, 2014 ▸, 2016 ▸; Korb *et al.*, 2017 ▸). The resulting ferrocenes are predominantly used as ligands in *C*,*C* cross-coupling catalysis (Schaarschmidt *et al.*, 2014 ▸; Jensen & Johannsen, 2003 ▸; Vinci *et al.*, 2009 ▸; Debono *et al.*, 2010 ▸; Karpus *et al.*, 2016 ▸), but also the introduction of ferrocenyl substituents by catalytic conversions is of rising inter­est (Hildebrandt *et al.*, 2011*a* ▸,*b* ▸; Speck *et al.*, 2015 ▸; Korb *et al.*, 2014*b* ▸). The introduction of electronically and sterically modified substrates requires the synthesis of the respective ferrocenes that bear groups suitable for oxidative additions or transmetalation reactions (Lehrich *et al.*, 2015 ▸; Speck *et al.*, 2014 ▸). In case of substrates that are sensitive towards a nucleophilic attack, *e.g*. acyl groups, the Suzuki–Miyaura instead of a Negishi reaction is commonly used, and hence requires the presence of a boronic acid functionality (Speck *et al.*, 2015 ▸). However, the acidic protons prevent a straightforward *ortho*-directed metalation, and additional reaction steps for the introduction and removal of protecting groups are required. Electrophilic aromatic substitution (S_E_Ar) reactions are also not suitable, since they usually give 1′- or 3-functionalized products (Rosenblum & Woodward, 1958 ▸).

Within our attempts to synthesize new electronically modified ferrocenes as substrates for Suzuki–Miyaura reactions, we herein present the synthesis and crystal structure of an *ortho*-functionalized ferrocenylboronic acid, obtained *via* S_E_Ar without using a protection group strategy for the acidic protons.
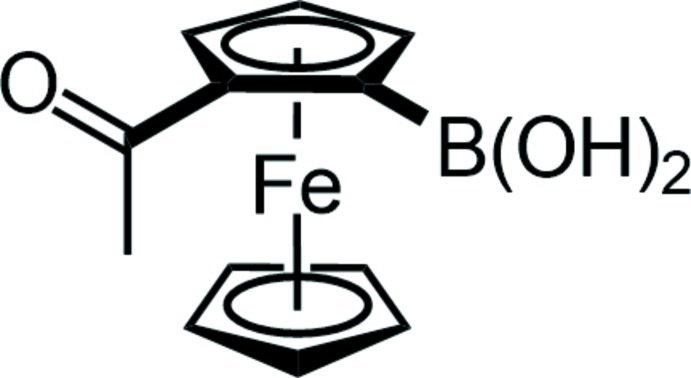


## Structural commentary   

**Figure 1:**
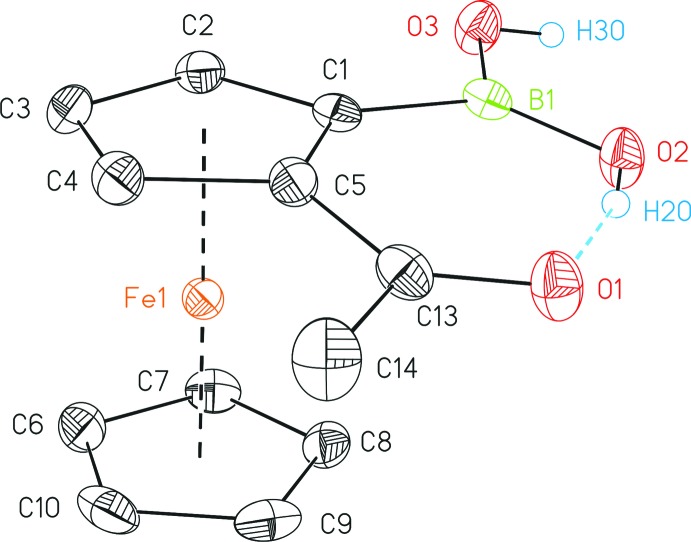
The mol­ecular structure of the title compound showing the intra­molecular hydrogen bond between the acetyl and the boronic acid functionalities. Displacement ellipsoids are drawn at the 50% probability level; C-bonded hydrogen atoms have been omitted for clarity.

The title compound crystallizes in the centrosymmetric space group *P*2_1_/*n* with one mol­ecule in the asymmetric unit (Fig. 1 ▸). An intra­molecular hydrogen bond between the oxygen atom of the acetyl group (O1) and the neighbouring hy­droxy group (O2) of the boronic acid functionality of 2.650 (2) Å (Table 1 ▸) is present. Therefore, both substituents are co-planar with each other [BO_2_⋯C_2_O = 2.9 (4)°]. The C=O distance of 1.233 (2) Å is neither affected by the involvement into this hydrogen bond, nor the presence of an *ortho* substituent and is therefore similar to unsubstituted acetyl ferrocene (Sato *et al.*, 1984 ▸).

With regard to the C_5_H_3_ plane of the ferrocenyl backbone, both substituents reveal a slight *endo*-bending of 7.0 (3)° (C_5_H_3_⋯C_2_O) and 9.5 (3)° (C_5_H_3_⋯BO_2_). The ferrocenyl backbone exhibits an eclipsed conformation (C1—*Cg*—*Cg*—C8 = 8.21 (14)°; *Cg* is the centroid of the respective cyclo­penta­dienyl ring) and a tilt angle of 179.28 (2)°. The hydrogen atom at O3 is directed away from the ferrocenyl backbone and points to an adjacent mol­ecule.

## Supra­molecular features   

**Figure 2:**
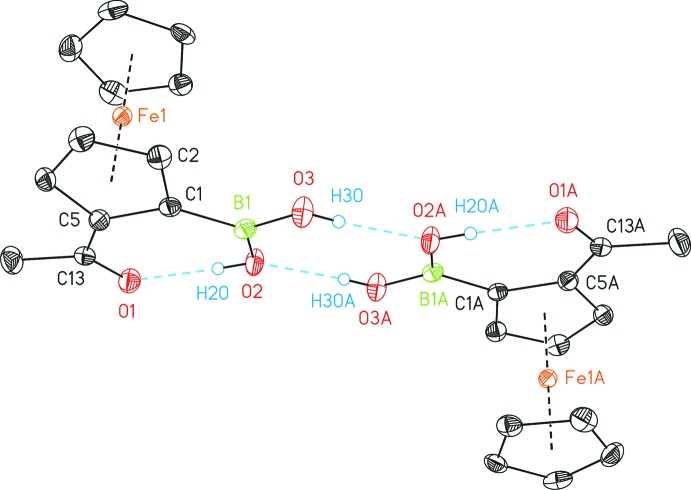
Intra- and inter­molecular hydrogen bonds within the dimer, with displacement ellipsoids drawn at the 50% probability level. C-bonded hydrogen atoms have been omitted for clarity. [Symmetry code: (A) 1 − *x*, 1 − *y*, 1 − *z*.]

**Figure 3:**
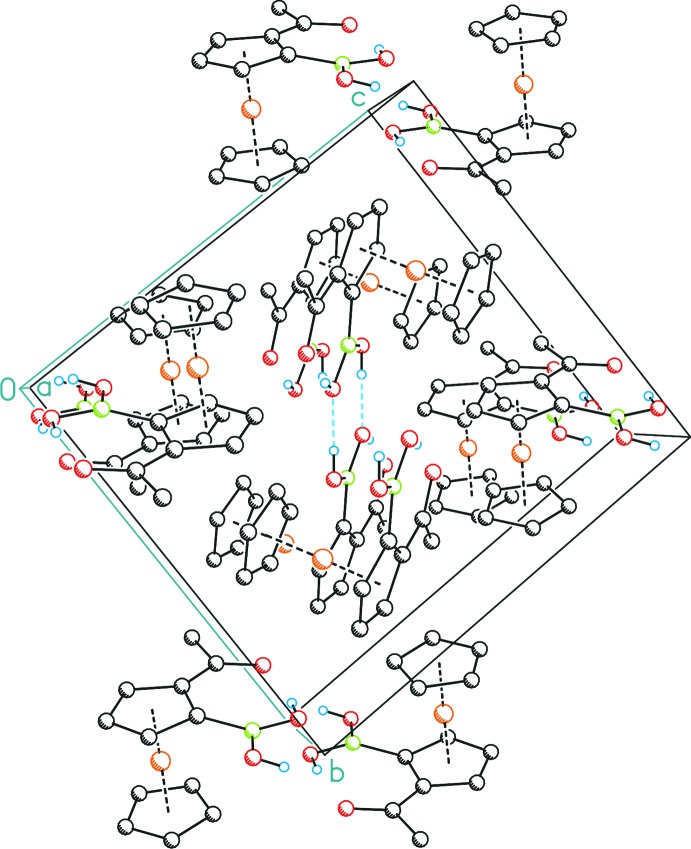
Unit cell of the title compound in a view along [100]. Hydrogen bonds are shown as pale-blue dashed lines; displacement ellipsoids are drawn at the 50% probability level. C-bonded hydrogen atoms have been omitted for clarity.

In addition to the aforementioned intra­molecular hydrogen bond between O1 and O2, the latter atom is also involved as an acceptor of an inter­molecular hydrogen bond with the second hy­droxy group (O3) of an adjacent boronic acid functionality of 2.744 (2) Å (Fig. 2 ▸, Table 1 ▸). The resulting dimer is centrosymmetric with the inversion center located at the middle of the eight-membered ring formed by the two boronic acid functionalities. Therefore, both ferrocenyl moieties are positioned *anti* with regard to the central B_2_O_4_ plane. Hence, a racemic mixture of both enanti­omers crystallized, giving the *R_p_*/*S_p_*-configured, *i.e. meso* diastereomer if the dimer is considered as one supra­molecular entity. The respective *racem* configuration (*R_p_*/*R_p_* or *S_p_*/*S_p_*) is not present within the packing (Fig. 3 ▸).

The B—O bond lengths involving O3 [1.356 (3) Å] and O2 [1.362 (3) Å] are similar, although the latter also acts as a hydrogen-bond acceptor, in contrast to O3.

A short contact of 4.6807 (14) Å between a C_5_H_3_ and a C_5_H_5_ ring does not show a perpendicular positioning of the two groups (*β* = 25°) and therefore does not fit the criteria for a *T*-shaped π–π inter­action (Sinnokrot *et al.*, 2002 ▸). However, weak C—H⋯O inter­actions between aromatic H atoms and the carbonyl O1 atom and a boronic acid O atom (O3) consolidate the crystal packing (Table 1 ▸).

## Database survey   

Besides ferrocenyl boronic acid (Bresner *et al.*, 2004 ▸) and acetyl ferrocene (Sato *et al.*, 1984 ▸) that are frequently used in general, other *ortho*-substituted analogues are sparsely described.

Crystal structures of acetyl­ferrocenes bearing additional *ortho*-substituents are limited to a few examples, *e.g*. with PPh_2_ (Torres *et al.*, 2011 ▸), iodine (Ferber *et al.*, 2007 ▸) and a ferrocenylmethyl group (Xie *et al.*, 2011 ▸) as the sole second substituent. In contrast, carbonyl, *i.e*. formyl or acyl groups, are more common, *e.g*. in ferrocenoyl methyl­ferrocene (Enders *et al.*, 2003 ▸).

Functionalized ferrocenylboronic acids are usually reported together with their protected 1,3,2-dioxaborolane derivatives. As *ortho*-substituents, di*iso*propyl­carbamoyl (Batsanov *et al.*, 2007 ▸) and di­methyl­carbamoyl (Norrild & Søtofte, 2001 ▸), together with their respective amino­methyl derivatives (Batsanov *et al.*, 2007 ▸; Norrild & Søtofte, 2001 ▸) have structurally been described. Heterocycles, such as imidazolidone (Metallinos *et al.*, 2012 ▸) are also known as *ortho*-substituents for ferrocenyl derivatives.

In case of non-ferrocenyl-based aromatics, the 2-C(O)CH_3_-1-B(OH)_2_ substitution pattern is solely reported for the benzene core (Ganguly *et al.*, 2003 ▸). In contrast to the title compound, the boronic acid functionality is rotated out of co-planarity with the benzene core and the acetyl group by 78.2 and 77.7°, respectively.

For *ortho*-carbonyl groups in general, the involvement of the boronic acid functionality in inter- and intra­molecular hydrogen bonds, similar to the title compound, is a common feature (Yan *et al.*, 2003 ▸; Luliński *et al.*, 2007 ▸; Durka *et al.*, 2014 ▸; Madura *et al.*, 2015 ▸).

## Synthesis and crystallization   

Ferroceneboronic acid (0.5 g, 2.175 mmol) was suspended in acetic anhydride (10 ml). To this suspension BF_3_·OEt_2_ (0.40 ml, 3.15 mmol) was added in a single portion. The reaction mixture was stirred for 30 min at ambient temperature. Afterwards, the mixture was poured into ice and was stirred for 10 minutes. A KOH solution (9 *M*, 10 ml) was added in a single portion following a neutralization with K_2_CO_3_ until the CO_2_ evolution stopped. The mixture was extracted with di­chloro­methane (3×20 ml) and the organic phase was dried over MgSO_4_. The volatiles were removed in vacuum (1 mbar). The crude material obtained was purified by flash chromatography on silica using a 4/1 (*v*/*v*) diethyl ether/di­chloro­methane mixture. The title compound was isolated as a brown solid. Yield: 75 mg (0.28 mmol, 13% based on ferroceneboronic acid).

IR data (KBr, ν/cm^−1^): 3357 (*w*), 2925 (*m*), 2855 (*m*), 1685 (*m*), 1654 (*s*), 1647 (*m*), 1636 (*s*), 1618 (*s*), 1578 (*m*), 1559 (*m*), 1522 (*m*), 1507 (*m*), 1457 (*s*); 1419 (*s*), 1411 (*s*), 1374 (*s*), 1354 (*s*), 1345 (*s*); 1318 (*m*), 1247 (*m*), 1207 (*m*), 1134 (*m*), 1106 (*m*), 1094 (*m*), 1045 (*m*), 1001 (*w*), 924 (*w*), 873 (*w*), 862 (*w*), 785 (*w*), 668 (*m*), 642 (*w*). ^1^H NMR (CDCl_3_, δ): 2.49 (*s*, 3H, CH_3_), 4.23 (*s*, 5H, C_5_H_5_), 4.78 (*t*, *J*_HH_ = 2.6 Hz, 1H, C_5_H_3_), 4.92 (*dd*, *J*_HH_ = 2.6 Hz, 1.3 Hz, 1H, C_5_H_3_), 5.01 (*dd*, *J*_HH_ = 2.6 Hz, 1.3 Hz, 1H, C_5_H_3_), 7.38 (*br s*, 2H, B(OH)_2_). ^13^C{^1^H} NMR (CDCl_3_, δ): 28.1 (CH_3_), 71.1 (C_5_H_5_), 76.1 (C_5_H_3_), 77.2 (C_5_H_3_), 80.1 (C_5_H_3_), 81.1 (C_5_H_3_), 81.8 (C_5_H_3_), 208.1 (CO). HRMS (ESI–TOF, *m*/*z*). calculated for C_12_H_13_BFeO_3_ 272.0304, found 272.0320 [*M*]^+^.

Crystals suitable for X-ray crystallography were obtained from evaporation of a saturated di­chloro­methane solution at ambient temperature.

## Refinement   

Crystal data, data collection and structure refinement detail are summarized in Table 2 ▸. C-bound H atoms were placed in calculated positions and constrained to ride on their parent atoms with *U*_iso_(H) = 1.2*U*_eq_(C) and a C—H distance of 0.93 Å for aromatic and *U*_iso_(H) = 1.5*U*_eq_(C) and a C—H distance of 0.96 Å for methyl H atoms, with their torsion angle derived from the residual electron density. The hy­droxy hydrogen atoms were located from difference-Fourier maps but were treated with idealized geometry with *U*_iso_(H) = 1.5*U*_eq_(O), an O—H distance of 0.82 Å and a torsion angle derived from the residual electron density.

## Supplementary Material

Crystal structure: contains datablock(s) I. DOI: 10.1107/S2056989019001178/wm5476sup1.cif

Structure factors: contains datablock(s) I. DOI: 10.1107/S2056989019001178/wm5476Isup2.hkl

CCDC reference: 1892711

Additional supporting information:  crystallographic information; 3D view; checkCIF report

## Figures and Tables

**Table 1:** Hydrogen-bond geometry (Å, °)

*D*—H⋯*A*	*D*—H	H⋯*A*	*D*⋯*A*	*D*—H⋯*A*
O2—H2*O*⋯O1	0.82	1.85	2.650 (2)	166
O3—H3*O*⋯O2^i^	0.82	1.94	2.744 (2)	168
C9—H9⋯O3^ii^	0.93	2.45	3.308 (3)	154
C10—H10⋯O1^iii^	0.93	2.53	3.404 (3)	156

Symmetry codes: (i) 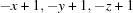; (ii) 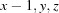; (iii) 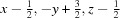.

**Table 2:** Experimental details

Crystal data	
Chemical formula	[Fe(C_5_H_5_)(C_7_H_8_BO_3_)]
*M*_r_	271.88
Crystal system, space group	Monoclinic, *P*2_1_/*n*
Temperature (K)	116
*a*, *b*, *c* (Å)	7.7627 (3), 11.7335 (5), 12.7969 (5)
β (°)	98.527 (4)
*V* (Å^3^)	1152.70 (8)
*Z*	4
Radiation type	Mo *K*α
μ (mm^−1^)	1.30
Crystal size (mm)	0.40 × 0.25 × 0.20

Data collection	
Diffractometer	Oxford Gemini S
Absorption correction	Multi-scan (*CrysAlis PRO*; Rigaku OD, 2015 ▸)
*T*_min_, *T*_max_	0.868, 1.000
No. of measured, independent and observed [*I* > 2σ(*I*)] reflections	4556, 2406, 2108
*R*_int_	0.019
(sin θ/λ)_max_ (Å^−1^)	0.667

Refinement	
*R*[*F* ^2^ > 2σ(*F* ^2^)], *wR*(*F* ^2^), *S*	0.030, 0.071, 1.08
No. of reflections	2406
No. of parameters	157
H-atom treatment	H-atom parameters constrained
Δρ_max_, Δρ_min_ (e Å^−3^)	0.37, −0.28

Computer programs: *CrysAlis CCD* and *CrysAlis RED* (Oxford Diffraction, 2014 ▸), *SHELXT2013* (Sheldrick, 2015*a* ▸), *SHELXL2013* (Sheldrick, 2015*b* ▸), *ORTEP-3 for Windows* and *WinGX* (Farrugia, 2012 ▸), *SHELXTL* (Sheldrick, 2008 ▸) and *publCIF* (Westrip, 2010 ▸).

## supplementary crystallographic information

### Crystal data

**Table:**

[Fe(C_5_H_5_)(C_7_H_8_BO_3_)]	*F*(000) = 560
*M**_r_* = 271.88	*D*_x_ = 1.567 Mg m^−^^3^
Monoclinic, *P*2_1_/*n*	Mo *K*α radiation, λ = 0.71073 Å
*a* = 7.7627 (3) Å	Cell parameters from 2357 reflections
*b* = 11.7335 (5) Å	θ = 3.7–28.0°
*c* = 12.7969 (5) Å	µ = 1.30 mm^−^^1^
β = 98.527 (4)°	*T* = 116 K
*V* = 1152.70 (8) Å^3^	Block, orange
*Z* = 4	0.40 × 0.25 × 0.20 mm

### Data collection

**Table:**

Oxford Gemini S diffractometer	2108 reflections with *I* > 2σ(*I*)
Radiation source: fine-focus sealed tube	*R*_int_ = 0.019
Graphite monochromator	θ_max_ = 28.3°, θ_min_ = 3.2°
ω scans	*h* = −9→10
Absorption correction: multi-scan (CrysAlis PRO; Rigaku OD, 2015)	*k* = −13→15
*T*_min_ = 0.868, *T*_max_ = 1.000	*l* = −16→16
4556 measured reflections	2 standard reflections every 50 reflections
2406 independent reflections	intensity decay: none

### Refinement

**Table:**

Refinement on *F*^2^	0 restraints
Least-squares matrix: full	Hydrogen site location: inferred from neighbouring sites
*R*[*F*^2^ > 2σ(*F*^2^)] = 0.030	H-atom parameters constrained
*wR*(*F*^2^) = 0.071	*w* = 1/[σ^2^(*F*_o_^2^) + (0.028*P*)^2^ + 0.4245*P*] where *P* = (*F*_o_^2^ + 2*F*_c_^2^)/3
*S* = 1.08	(Δ/σ)_max_ = 0.001
2406 reflections	Δρ_max_ = 0.37 e Å^−^^3^
157 parameters	Δρ_min_ = −0.28 e Å^−^^3^

### Special details

**Table:**

Geometry. All esds (except the esd in the dihedral angle between two l.s. planes) are estimated using the full covariance matrix. The cell esds are taken into account individually in the estimation of esds in distances, angles and torsion angles; correlations between esds in cell parameters are only used when they are defined by crystal symmetry. An approximate (isotropic) treatment of cell esds is used for estimating esds involving l.s. planes.
Refinement. Refinement of F^2^ against ALL reflections. The weighted R factor wR and goodness of fit S are based on F^2^, conventional R factors R are based on F, with F set to zero for negative F^2^. The threshold expression of F^2^ > 2σ(F^2^) is used only for calculating R factors(gt) etc. and is not relevant to the choice of reflections for refinement. R factors based on F^2^ are statistically about twice as large as those based on F, and R factors based on ALL data will be even larger.

### Fractional atomic coordinates and isotropic or equivalent isotropic displacement parameters (Å^2^)

**Table:**

	*x*	*y*	*z*	*U*_iso_*/*U*_eq_	
C1	0.2362 (2)	0.71679 (17)	0.35395 (14)	0.0159 (4)	
C2	0.2734 (3)	0.75344 (17)	0.25306 (15)	0.0189 (4)	
H2	0.3701	0.7314	0.2229	0.023*	
C3	0.1414 (3)	0.82799 (17)	0.20593 (16)	0.0202 (4)	
H3	0.1361	0.8621	0.1399	0.024*	
C4	0.0188 (3)	0.84184 (17)	0.27622 (15)	0.0193 (4)	
H4	−0.0804	0.8872	0.2649	0.023*	
C5	0.0743 (3)	0.77358 (17)	0.36840 (14)	0.0174 (4)	
C6	−0.0701 (3)	0.60243 (19)	0.09838 (15)	0.0243 (5)	
H6	−0.0624	0.6319	0.0318	0.029*	
C7	0.0489 (3)	0.52589 (18)	0.15604 (15)	0.0211 (4)	
H7	0.1485	0.4961	0.1339	0.025*	
C8	−0.0103 (3)	0.50261 (18)	0.25363 (15)	0.0208 (4)	
H8	0.0434	0.4547	0.3066	0.025*	
C9	−0.1650 (3)	0.56502 (19)	0.25598 (17)	0.0249 (5)	
H9	−0.2307	0.5658	0.3111	0.030*	
C10	−0.2028 (3)	0.6261 (2)	0.16011 (17)	0.0271 (5)	
H10	−0.2982	0.6736	0.1410	0.033*	
C13	−0.0314 (3)	0.75364 (18)	0.45290 (15)	0.0191 (4)	
C14	−0.1979 (3)	0.8197 (2)	0.44975 (17)	0.0286 (5)	
H14A	−0.2495	0.8027	0.5117	0.043*	
H14B	−0.2771	0.7988	0.3878	0.043*	
H14C	−0.1735	0.8998	0.4478	0.043*	
B1	0.3493 (3)	0.6251 (2)	0.42048 (17)	0.0180 (5)	
O1	0.01228 (19)	0.68590 (13)	0.52571 (11)	0.0242 (3)	
O2	0.30364 (18)	0.57462 (13)	0.50825 (10)	0.0224 (3)	
H2O	0.2091	0.5995	0.5190	0.034*	
O3	0.50175 (17)	0.59706 (13)	0.38717 (11)	0.0237 (3)	
H3O	0.5472	0.5439	0.4227	0.036*	
Fe1	0.03493 (3)	0.67352 (2)	0.23909 (2)	0.01438 (10)	

### Atomic displacement parameters (Å^2^)

**Table:**

	*U*^11^	*U*^22^	*U*^33^	*U*^12^	*U*^13^	*U*^23^
C1	0.0146 (9)	0.0161 (10)	0.0164 (9)	−0.0020 (8)	0.0000 (7)	−0.0028 (8)
C2	0.0174 (10)	0.0184 (10)	0.0211 (10)	−0.0030 (9)	0.0032 (8)	0.0000 (9)
C3	0.0238 (11)	0.0167 (10)	0.0198 (10)	−0.0035 (9)	0.0022 (8)	0.0045 (9)
C4	0.0216 (10)	0.0140 (10)	0.0218 (10)	0.0025 (8)	0.0010 (8)	−0.0010 (9)
C5	0.0191 (10)	0.0151 (10)	0.0173 (9)	0.0011 (8)	0.0006 (8)	−0.0004 (8)
C6	0.0308 (12)	0.0261 (12)	0.0140 (9)	−0.0074 (10)	−0.0029 (8)	−0.0015 (9)
C7	0.0218 (10)	0.0192 (11)	0.0228 (10)	−0.0017 (9)	0.0053 (8)	−0.0094 (9)
C8	0.0256 (11)	0.0151 (10)	0.0214 (10)	−0.0030 (9)	0.0020 (9)	0.0002 (9)
C9	0.0201 (11)	0.0265 (12)	0.0297 (11)	−0.0095 (9)	0.0096 (9)	−0.0062 (10)
C10	0.0176 (10)	0.0245 (12)	0.0358 (12)	−0.0013 (10)	−0.0074 (9)	−0.0046 (10)
C13	0.0192 (10)	0.0200 (11)	0.0174 (9)	0.0015 (9)	0.0006 (8)	−0.0052 (9)
C14	0.0276 (12)	0.0328 (13)	0.0270 (11)	0.0116 (10)	0.0096 (10)	0.0015 (10)
B1	0.0160 (11)	0.0185 (11)	0.0186 (10)	−0.0014 (10)	−0.0001 (9)	−0.0027 (10)
O1	0.0239 (8)	0.0309 (9)	0.0184 (7)	0.0073 (7)	0.0048 (6)	0.0034 (7)
O2	0.0183 (7)	0.0286 (9)	0.0208 (7)	0.0084 (7)	0.0048 (6)	0.0071 (7)
O3	0.0190 (7)	0.0253 (9)	0.0276 (8)	0.0054 (7)	0.0059 (6)	0.0084 (7)
Fe1	0.01391 (16)	0.01475 (16)	0.01429 (15)	0.00022 (11)	0.00151 (11)	0.00008 (11)

### Geometric parameters (Å, º)

**Table:**

C1—C2	1.430 (3)	C7—Fe1	2.043 (2)
C1—C5	1.459 (3)	C7—H7	0.9300
C1—B1	1.560 (3)	C8—C9	1.411 (3)
C1—Fe1	2.0443 (18)	C8—Fe1	2.049 (2)
C2—C3	1.413 (3)	C8—H8	0.9300
C2—Fe1	2.059 (2)	C9—C10	1.414 (3)
C2—H2	0.9300	C9—Fe1	2.043 (2)
C3—C4	1.412 (3)	C9—H9	0.9300
C3—Fe1	2.062 (2)	C10—Fe1	2.045 (2)
C3—H3	0.9300	C10—H10	0.9300
C4—C5	1.438 (3)	C13—O1	1.233 (2)
C4—Fe1	2.040 (2)	C13—C14	1.503 (3)
C4—H4	0.9300	C14—H14A	0.9600
C5—C13	1.470 (3)	C14—H14B	0.9600
C5—Fe1	2.0150 (19)	C14—H14C	0.9600
C6—C7	1.415 (3)	B1—O3	1.356 (3)
C6—C10	1.416 (3)	B1—O2	1.362 (3)
C6—Fe1	2.0414 (19)	O2—H2O	0.8200
C6—H6	0.9300	O3—H3O	0.8200
C7—C8	1.420 (3)		
			
C2—C1—C5	105.66 (17)	O1—C13—C5	122.50 (18)
C2—C1—B1	121.86 (17)	O1—C13—C14	119.45 (18)
C5—C1—B1	132.22 (17)	C5—C13—C14	118.04 (17)
C2—C1—Fe1	70.16 (11)	C13—C14—H14A	109.5
C5—C1—Fe1	67.87 (10)	C13—C14—H14B	109.5
B1—C1—Fe1	121.98 (14)	H14A—C14—H14B	109.5
C3—C2—C1	109.94 (18)	C13—C14—H14C	109.5
C3—C2—Fe1	70.07 (11)	H14A—C14—H14C	109.5
C1—C2—Fe1	69.05 (11)	H14B—C14—H14C	109.5
C3—C2—H2	125.0	O3—B1—O2	119.99 (19)
C1—C2—H2	125.0	O3—B1—C1	116.25 (18)
Fe1—C2—H2	127.5	O2—B1—C1	123.75 (18)
C4—C3—C2	108.30 (18)	B1—O2—H2O	109.5
C4—C3—Fe1	69.01 (12)	B1—O3—H3O	109.5
C2—C3—Fe1	69.84 (11)	C5—Fe1—C4	41.54 (8)
C4—C3—H3	125.9	C5—Fe1—C6	162.67 (9)
C2—C3—H3	125.9	C4—Fe1—C6	124.81 (9)
Fe1—C3—H3	126.9	C5—Fe1—C7	155.19 (8)
C3—C4—C5	108.07 (18)	C4—Fe1—C7	162.14 (8)
C3—C4—Fe1	70.72 (12)	C6—Fe1—C7	40.53 (8)
C5—C4—Fe1	68.32 (11)	C5—Fe1—C9	107.54 (8)
C3—C4—H4	126.0	C4—Fe1—C9	120.33 (9)
C5—C4—H4	126.0	C6—Fe1—C9	68.20 (9)
Fe1—C4—H4	126.6	C7—Fe1—C9	68.08 (8)
C4—C5—C1	108.02 (17)	C5—Fe1—C1	42.11 (8)
C4—C5—C13	124.06 (18)	C4—Fe1—C1	70.05 (8)
C1—C5—C13	127.32 (17)	C6—Fe1—C1	153.94 (9)
C4—C5—Fe1	70.14 (11)	C7—Fe1—C1	119.38 (8)
C1—C5—Fe1	70.02 (11)	C9—Fe1—C1	126.18 (8)
C13—C5—Fe1	118.56 (14)	C5—Fe1—C10	125.46 (9)
C7—C6—C10	107.85 (18)	C4—Fe1—C10	107.24 (9)
C7—C6—Fe1	69.81 (11)	C6—Fe1—C10	40.54 (9)
C10—C6—Fe1	69.88 (11)	C7—Fe1—C10	68.05 (9)
C7—C6—H6	126.1	C9—Fe1—C10	40.46 (9)
C10—C6—H6	126.1	C1—Fe1—C10	163.81 (9)
Fe1—C6—H6	125.8	C5—Fe1—C8	120.19 (8)
C6—C7—C8	108.03 (18)	C4—Fe1—C8	155.41 (8)
C6—C7—Fe1	69.65 (12)	C6—Fe1—C8	68.21 (8)
C8—C7—Fe1	69.91 (11)	C7—Fe1—C8	40.60 (8)
C6—C7—H7	126.0	C9—Fe1—C8	40.32 (8)
C8—C7—H7	126.0	C1—Fe1—C8	107.50 (8)
Fe1—C7—H7	126.0	C10—Fe1—C8	67.96 (9)
C9—C8—C7	107.85 (18)	C5—Fe1—C2	68.80 (8)
C9—C8—Fe1	69.61 (12)	C4—Fe1—C2	67.92 (8)
C7—C8—Fe1	69.49 (12)	C6—Fe1—C2	119.92 (8)
C9—C8—H8	126.1	C7—Fe1—C2	108.35 (8)
C7—C8—H8	126.1	C9—Fe1—C2	164.08 (8)
Fe1—C8—H8	126.4	C1—Fe1—C2	40.79 (7)
C8—C9—C10	108.23 (19)	C10—Fe1—C2	154.21 (9)
C8—C9—Fe1	70.06 (12)	C8—Fe1—C2	126.99 (8)
C10—C9—Fe1	69.85 (12)	C5—Fe1—C3	68.91 (8)
C8—C9—H9	125.9	C4—Fe1—C3	40.27 (8)
C10—C9—H9	125.9	C6—Fe1—C3	107.30 (8)
Fe1—C9—H9	125.8	C7—Fe1—C3	125.75 (8)
C9—C10—C6	108.05 (19)	C9—Fe1—C3	154.59 (9)
C9—C10—Fe1	69.69 (12)	C1—Fe1—C3	69.07 (8)
C6—C10—Fe1	69.58 (12)	C10—Fe1—C3	119.80 (9)
C9—C10—H10	126.0	C8—Fe1—C3	163.37 (8)
C6—C10—H10	126.0	C2—Fe1—C3	40.09 (8)
Fe1—C10—H10	126.3		
			
C5—C1—C2—C3	−0.7 (2)	C10—C6—C7—Fe1	−59.77 (15)
B1—C1—C2—C3	174.20 (18)	C6—C7—C8—C9	−0.2 (2)
Fe1—C1—C2—C3	58.18 (14)	Fe1—C7—C8—C9	59.26 (14)
C5—C1—C2—Fe1	−58.86 (13)	C6—C7—C8—Fe1	−59.45 (14)
B1—C1—C2—Fe1	116.02 (18)	C7—C8—C9—C10	0.5 (2)
C1—C2—C3—C4	0.9 (2)	Fe1—C8—C9—C10	59.65 (15)
Fe1—C2—C3—C4	58.44 (14)	C7—C8—C9—Fe1	−59.18 (14)
C1—C2—C3—Fe1	−57.58 (14)	C8—C9—C10—C6	−0.6 (2)
C2—C3—C4—C5	−0.7 (2)	Fe1—C9—C10—C6	59.21 (15)
Fe1—C3—C4—C5	58.27 (14)	C8—C9—C10—Fe1	−59.78 (15)
C2—C3—C4—Fe1	−58.96 (14)	C7—C6—C10—C9	0.5 (2)
C3—C4—C5—C1	0.3 (2)	Fe1—C6—C10—C9	−59.28 (15)
Fe1—C4—C5—C1	60.03 (14)	C7—C6—C10—Fe1	59.73 (14)
C3—C4—C5—C13	−171.44 (18)	C4—C5—C13—O1	172.7 (2)
Fe1—C4—C5—C13	−111.68 (19)	C1—C5—C13—O1	2.7 (3)
C3—C4—C5—Fe1	−59.77 (14)	Fe1—C5—C13—O1	88.4 (2)
C2—C1—C5—C4	0.3 (2)	C4—C5—C13—C14	−7.8 (3)
B1—C1—C5—C4	−173.9 (2)	C1—C5—C13—C14	−177.86 (19)
Fe1—C1—C5—C4	−60.11 (14)	Fe1—C5—C13—C14	−92.1 (2)
C2—C1—C5—C13	171.61 (19)	C2—C1—B1—O3	11.5 (3)
B1—C1—C5—C13	−2.5 (4)	C5—C1—B1—O3	−175.2 (2)
Fe1—C1—C5—C13	111.3 (2)	Fe1—C1—B1—O3	96.7 (2)
C2—C1—C5—Fe1	60.36 (13)	C2—C1—B1—O2	−169.19 (19)
B1—C1—C5—Fe1	−113.8 (2)	C5—C1—B1—O2	4.1 (4)
C10—C6—C7—C8	−0.2 (2)	Fe1—C1—B1—O2	−84.0 (2)
Fe1—C6—C7—C8	59.61 (14)		

### Hydrogen-bond geometry (Å, º)

**Table:**

*D*—H···*A*	*D*—H	H···*A*	*D*···*A*	*D*—H···*A*
O2—H2*O*···O1	0.82	1.85	2.650 (2)	166
O3—H3*O*···O2^i^	0.82	1.94	2.744 (2)	168
C9—H9···O3^ii^	0.93	2.45	3.308 (3)	154
C10—H10···O1^iii^	0.93	2.53	3.404 (3)	156

Symmetry codes: (i) −*x*+1, −*y*+1, −*z*+1; (ii) *x*−1, *y*, *z*; (iii) *x*−1/2, −*y*+3/2, *z*−1/2.

## References

[bb1] Batsanov, A. S., Hérault, D., Howard, J. A. K., Patrick, L. G. F., Probert, M. R. & Whiting, A. (2007). *Organometallics*, **26**, 2414–2419.

[bb2] Bresner, C., Aldridge, S., Fallis, I. A. & Ooi, L.-L. (2004). *Acta Cryst.* E**60**, m441–m443.

[bb3] Debono, N., Labande, A., Manoury, E., Daran, J.-C. & Poli, R. (2010). *Organometallics*, **29**, 1879–1882.

[bb4] Durka, K., Górska, A., Kliś, T., Serwatowski, J. & Woźniak, K. (2014). *Tetrahedron Lett.* **55**, 1234–1238.

[bb5] Enders, D., Klumpen, T. & Raabe, G. (2003). *Synlett*, pp. 1198–1200.

[bb6] Farrugia, L. J. (2012). *J. Appl. Cryst.* **45**, 849–854.

[bb7] Ferber, B., Top, S., Herson, P. & Jaouen, G. (2007). *Organometallics*, **26**, 1686–1691.

[bb8] Ganguly, A., Meyers, C. Y. & Robinson, P. D. (2003). *Acta Cryst.* E**59**, o759–o761.

[bb9] Hildebrandt, A., Schaarschmidt, D., Claus, R. & Lang, H. (2011*a*). *Inorg. Chem.* **50**, 10623–10632. 10.1021/ic200926z 21957943

[bb10] Hildebrandt, A., Schaarschmidt, D. & Lang, H. (2011*b*). *Organometallics*, **30**, 556–563.

[bb11] Jensen, J. F. & Johannsen, M. (2003). *Org. Lett.* **5**, 3025–3028. 10.1021/ol034943n 12916972

[bb12] Karpus, A., Yesypenko, O., Boiko, V., Poli, R., Daran, J.-C., Voitenko, Z., Kalchenko, V. & Manoury, E. (2016). *Eur. J. Org. Chem.* pp. 3386–3394. 10.1021/acs.joc.7b02312 29323909

[bb13] Korb, M. & Lang, H. (2014). *Organometallics*, **33**, 6643–6659.

[bb14] Korb, M. & Lang, H. (2016). *Inorg. Chem. Commun.* **72**, 30–32.

[bb15] Korb, M., Lehrich, S. W. & Lang, H. (2017). *J. Org. Chem.* **82**, 3102–3124. 10.1021/acs.joc.7b00030 28266218

[bb16] Korb, M., Pfaff, U., Hildebrandt, A., Rüffer, T. & Lang, H. (2014*b*). *Eur. J. Inorg. Chem.* pp. 1051–1061.

[bb17] Korb, M., Schaarschmidt, D. & Lang, H. (2014*a*). *Organometallics*, **33**, 2099–2108.

[bb18] Lehrich, S. W., Hildebrandt, A., Korb, M. & Lang, H. (2015). *J. Organomet. Chem.* **792**, 37–45.

[bb19] Luliński, S., Madura, I., Serwatowski, J., Szatyłowicz, H. & Zachara, J. (2007). *New J. Chem.* **31**, 144–154.

[bb20] Madura, I. D., Adamczyk-Woźniak, A. & Sporzyński, A. (2015). *J. Mol. Struct.* **1083**, 204–211.

[bb21] Metallinos, C., John, J., Zaifman, J. & Emberson, K. (2012). *Adv. Synth. Catal.* **354**, 602–606.

[bb22] Norrild, J. C. & Søtofte, I. (2001). *J. Chem. Soc. Perkin Trans. 2*, pp. 727–732.

[bb23] Oxford Diffraction (2014). *CrysAlis CCD* and *CrysAlis RED*. Oxford Diffraction, Abingdon, England.

[bb24] Rigaku OD (2015). *CrysAlis PRO*. Rigaku OD, Abingdon, England.

[bb25] Rosenblum, M. & Woodward, R. B. (1958). *J. Am. Chem. Soc.* **80**, 5443–5449.

[bb26] Sato, K., Katada, M., Sano, H. & Konno, M. (1984). *Bull. Chem. Soc. Jpn*, **57**, 2361–2365.

[bb27] Schaarschmidt, D., Grumbt, M., Hildebrandt, A. & Lang, H. (2014). *Eur. J. Org. Chem.* **2014**, 6676–6685.

[bb28] Schaarschmidt, D. & Lang, H. (2013). *Organometallics*, **32**, 5668–5704.

[bb29] Sheldrick, G. M. (2008). *Acta Cryst.* A**64**, 112–122. 10.1107/S0108767307043930 18156677

[bb30] Sheldrick, G. M. (2015*a*). *Acta Cryst.* A**71**, 3–8.

[bb31] Sheldrick, G. M. (2015*b*). *Acta Cryst.* C**71**, 3–8.

[bb32] Sinnokrot, M. O., Valeev, E. F. & Sherrill, C. D. (2002). *J. Am. Chem. Soc.* **124**, 10887–10893. 10.1021/ja025896h 12207544

[bb33] Speck, J. M., Korb, M., Rüffer, T., Hildebrandt, A. & Lang, H. (2014). *Organometallics*, **33**, 4813–4823.

[bb34] Speck, J. M., Korb, M., Schade, A., Spange, S. & Lang, H. (2015). *Organometallics*, **34**, 3788–3798.

[bb35] Torres, J., Sepúlveda, F., Carrión, M. C., Jalón, F. A., Manzano, B. R., Rodríguez, A. M., Zirakzadeh, A., Weissensteiner, W., Mucientes, A. E. & Peña, M. A. (2011). *Organometallics*, **30**, 3490–3503.

[bb36] Vinci, D., Martins, N., Saidi, O., Bacsa, J., Brigas, A. & Xiao, J. (2009). *Can. J. Chem.* **87**, 171–175. 10.1039/c2ob25187f 22505137

[bb37] Werner, G. & Butenschön, H. (2013). *Organometallics*, **32**, 5798–5809.

[bb38] Westrip, S. P. (2010). *J. Appl. Cryst.* **43**, 920–925.

[bb39] Xie, R.-J., Han, L.-M., Zhu, N., Hong, H.-L. & Suo, Q.-L. (2011). *J. Coord. Chem.* **64**, 3180–3188.

[bb40] Yan, H., Beatty, A. M. & Fehlner, T. P. (2003). *J. Am. Chem. Soc.* **125**, 16367–16382. 10.1021/ja038444t 14692779

